# Anti-Human α-Synuclein N-Terminal Peptide Antibody Protects against Dopaminergic Cell Death and Ameliorates Behavioral Deficits in an AAV-α-Synuclein Rat Model of Parkinson’s Disease

**DOI:** 10.1371/journal.pone.0116841

**Published:** 2015-02-06

**Authors:** Md Shahaduzzaman, Kevin Nash, Charles Hudson, Masroor Sharif, Bethany Grimmig, Xiaoyang Lin, Ge Bai, Hui Liu, Kenneth E. Ugen, Chuanhai Cao, Paula C. Bickford

**Affiliations:** 1 Center of Excellence for Aging & Brain Repair, Department of Neurosurgery and Brain Repair, University of South Florida Morsani College of Medicine, Tampa, Florida, 33612, United States of America; 2 Molecular Pharmacology and Physiology, University of South Florida Morsani College of Medicine, Tampa, Florida, 33612, United States of America; 3 James A. Haley Veterans Affairs Hospital, Research Service, Tampa, Florida, 33612, United States of America; 4 USF-Health Byrd Alzheimer’s Institute University of South Florida, Tampa, Florida, 33612, United States of America; 5 Dept. of Molecular Medicine, University of South Florida Morsani College of Medicine, Tampa, Florida, 33612, United States of America; 6 Center for Molecular Delivery, University of South Florida, Tampa, Florida, 33620, United States of America; 7 Dept. of Pharmaceutical Sciences, College of Pharmacy, University of South Florida, Tampa, Florida, 33612, United States of America

## Abstract

The protein α-synuclein (α-Syn) has a central role in the pathogenesis of Parkinson’s disease (PD) and immunotherapeutic approaches targeting this molecule have shown promising results. In this study, novel antibodies were generated against specific peptides from full length human α-Syn and evaluated for effectiveness in ameliorating α-Syn-induced cell death and behavioral deficits in an AAV-α-Syn expressing rat model of PD. Fisher 344 rats were injected with rAAV vector into the right substantia nigra (SN), while control rats received an AAV vector expressing green fluorescent protein (GFP). Beginning one week after injection of the AAV-α-Syn vectors, rats were treated intraperitoneally with either control IgG or antibodies against the N-terminal (AB1), or central region (AB2) of α-Syn. An unbiased stereological estimation of TH+, NeuN+, and OX6 (MHC-II) immunostaining revealed that the α-Syn peptide antibodies (AB1 and AB2) significantly inhibited α-Syn-induced dopaminergic cell (DA) and NeuN+ cell loss (one-way ANOVA (F (3, 30) = 5.8, *p* = 0.002 and (F (3, 29) = 7.92, *p* = 0.002 respectively), as well as decreasing the number of activated microglia in the ipsilateral SN (one-way ANOVA F = 14.09; p = 0.0003). Antibody treated animals also had lower levels of α-Syn in the ipsilateral SN (one-way ANOVA F (7, 37) = 9.786; p = 0.0001) and demonstrated a partial intermediate improvement of the behavioral deficits. Our data suggest that, in particular, an α-Syn peptide antibody against the N-terminal region of the protein can protect against DA neuron loss and, to some extent behavioral deficits. As such, these results may be a potential therapeutic strategy for halting the progression of PD.

## Introduction

Aggregrates of the brain protein alpha-synuclein (α-Syn) are generally considered to have a major role in the pathological development and progression of PD [1]. Active or passive immunotherapy directed against misfolded proteins associated with neurodegenerative diseases such as α-Syn for PD [2–4] and amyloid beta (Aβ) for Alzheimer’s disease (AD) therapy, have yielded promising results [5,6]. A number of clinical trials on immunotherapies against Aβ are now under way [7,8]. Several clinical studies have demonstrated the efficacy of immune-based approaches in lowering Aβ load in the brains of AD patients [9,10]. However, vaccine associated side effects such as meningoencephalitis and cerebral microhemorrhaging in the brains of some of the subjects in the Aβ vaccine trials have tempered the enthusiasm for this strategy [11–13]. Preclinical evidence has suggested that other misfolded proteins including hyperphosphorylated tau, prion proteins, huntington, TAR DNA-binding protein 43, and mutant superoxide dismutase 1 (SOD1) can also be targeted for immunotherapeutic strategies [14]. Evidence supporting immunotherapy against α-Syn as an experimental treatment option for PD comes from preclinical studies using different mouse models for PD [15,16]. Inhibition of α-Syn aggregation using small molecules, enhanced clearance of α-Syn through the lysosomal pathways, and decreased neuroinflammation are among the most prevalent therapeutic strategies being investigated [17]. However, targeting intracellular α-Syn protein continues to be a major challenge for immunotherapy due to the presence of diverse forms (i.e. oligomeric and phosphorylated) detected in human plasma and CSF [18,19]. Despite the scientific progress using immunotherapy against other neurogdegenerative diseases, only one clinical trial (AFFITOPE PD01A, NCT01568099) has been approved for PD to date.

Structurally, human α-Syn is an intrinsically disordered 140 amino acid long protein consisting of three distinct regions: an N-terminal region (residues 1–60) which forms a helical structure and interacts with the celllular membrane [20], a central highly aggregration-prone non-Aβ component region (residues 61–95) [21] and a C-terminal region (residues 96–140) that is highly enriched in acidic residues and prolines [22]. It has been demonstrated that immunotherapy with an antibody targeted against the C-terminus of α-Syn promoted clearance of this protein from neuronal cells in an α-Syn expressing transgenic PD mouse model [23]. Other researchers have demonstrated that these antibodies can enter the brain and reduce both intracellular and extracellular levels of α-Syn [24]. To date, there have not been any studies evaluating the potential efficacy of antibodies directed against the N-terminal region of α-Syn. It has been demonstrated that all three mutations of α-Syn, A30P, E46K and A53T, occur within the N-terminal region and are associated with inherited early-onset variants of PD. These mutants are able to accelerate α-Syn oligomerization and protofibrilar aggregation of this protein [25]. Thus, the identification of the interaction sites within the N-terminal regions with specific antibodies may provide a novel immunotherapeutic approach against PD.

Age has been determined to be a major risk factor for neurodegenerative diseases such as AD and PD. Of relevance as well is the observation that immune responses also decline with age which may potentially have an important role in the pathophysiology of neurodegenerative diseases. Importantly, in both human and animal models of PD α-Syn aggregation is accompanied by activation of both the innate and adaptive immune responses [26,27]. These include increased microglial activation as evidenced by enhanced MHCII expression [28], altered serum IgG production, [29] and infiltration of CD4 lymphocytes surrounding degenerating neurons [30]. Post-mortem studies of the brains of patients suffering from PD have consistently demonstrated microglial activation in the SN. It has been proposed that activated microglia promote α-Syn aggregation by perpetuating pro-inflammatory immune responses in PD brains through the generation of reactive oxygen species (ROS) and many other soluble factors, including chemokines and cytokines such as TNF-α, NFκB1,IL-15, RANTES, and IL-10 [31]. This ultimately leads to further neurodegeneration. However, it has also been demonstrated that immune responses against α-Syn can mediate the removal of this protein, to varying extents, from the brain.

It has been reported previously that anti-α-Syn monoclonal antibodies directed against the C-terminal of α-Syn enhanced the clearing of intracellular α-Syn aggregates [23,32]. Recently, a monoclonal antibody (Syn303) directed against N-terminus of α synuclein (amino acids 1–5) reduced propagation of synuclein fibrils in the ipsilateral frontal cortex, SNpc, and the amygdala [33]. The main goal of this study was to determine the potential protective effects of passive immunotherapy with an anti-α-Syn antibody directed against the N-terminus or central region of α-Syn. In contrast to active vaccination, passive immunotherapy has been demonstrated to have a regulatory effect on microglial equilibrium and may be a safer alternative to active immunization [34]. Our results demonstrated that these antibodies confer neural protection and ameliorate behavioral deficits by reducing the levels of α-Syn. The second goal of this study was to investigate the effects of the generated antibodies on PD-associated immune response impairment, notably on microglial homeostasis. On that point it was determined that the antibody treatments evaluated in this study considerably reduces the number of activated microglia, therefore preventing the progressive loss of DA from α-Syn mediated toxicity. From these results it is concluded that passive immunotherapy against the N-terminus of α-Syn may be a valid and useful therapeutic strategy against PD and warrants further examination.

## Methods

### Animals

**Fig 1:**
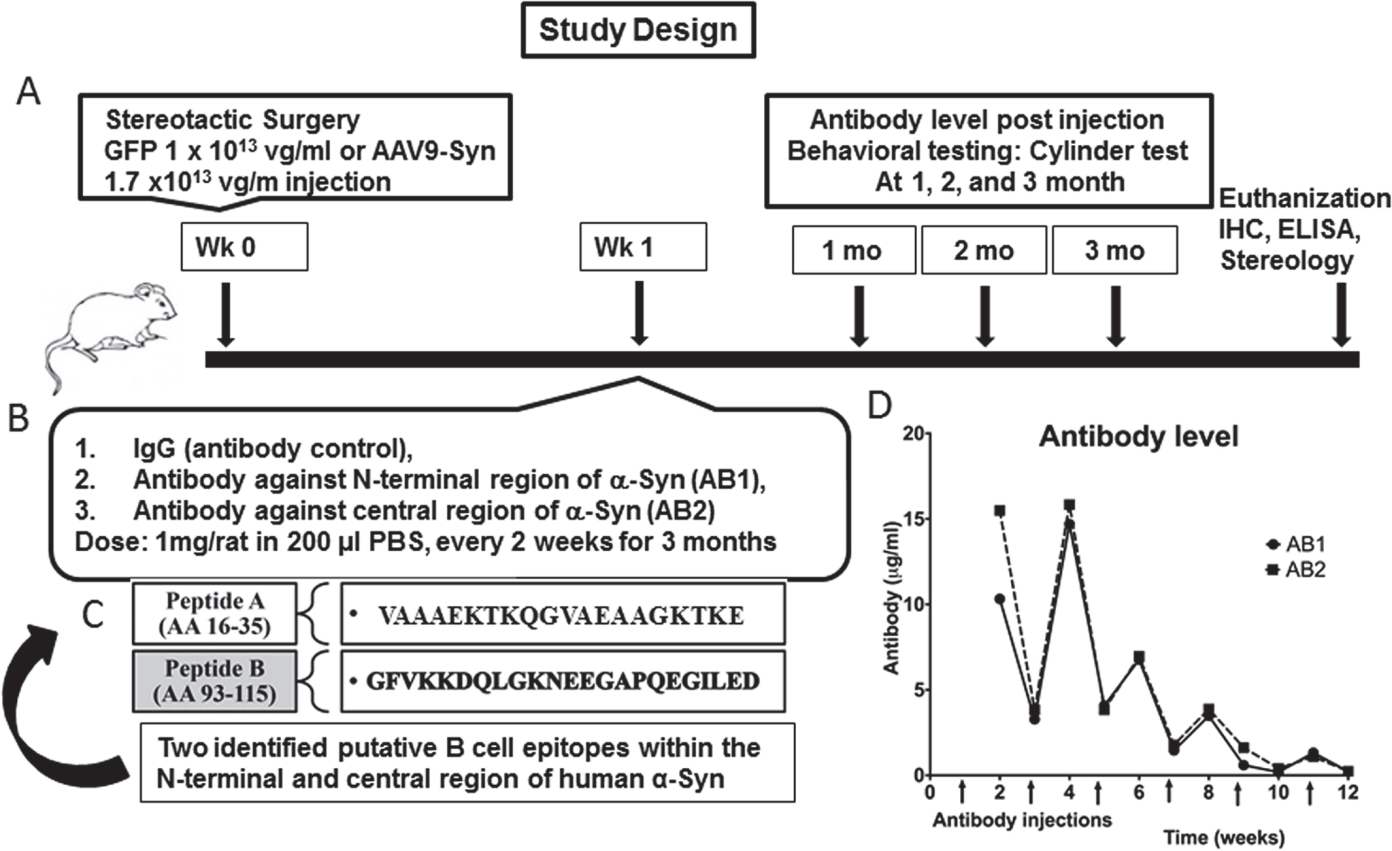
Study design to test the efficacy of anti- α-Syn antibody in rat AAV- α-Syn PD model. A schematic diagram depicting (A) the details on AAV9 concentrations used and the time frame for AAV-9 injections and the behavioral testing, (B) the timing of the first antibody injections and initial dose, as indicated in the methods section the dose of the AB injected was reduced over time (C) the sequence of the antibodies and (D) level of serum antibodies taken 1 week after injections and just before subsequent injections. This demonstrates that antibody levels remained high for the first month and then clearance increased after 6 weeks. Times of injections are indicated by the arrows on the bottom of the graph.

Animal studies were approved by the University of South Florida’s Institutional Animal Care and Use Committee. All animal procedures were performed according to the NIH guidelines for the care and use of animals. Fisher 344 male rats (Harlan) were pair-housed in a 12 hour light/dark cycle with access to water and food *ad libitum*. Rats *w*ere randomly assigned to either 1) AAV-α-Syn + IgG (non-immune antibody control); 2) AAV-α-Syn-AB1 (treatment 1); 3) AAV-α-Syn-AB2 (treatment 2); or 4) AAV-GFP (negative control) groups (**Fig. 1**)

### Vector Construction and AAV-α-Syn Administration through Stereotaxic Surgery

A recombinant adeno-associated viral vector (rAAV) serotype 9 expressing human wild type (WT) α-Syn or green fluorescent protein (GFP) under control of the CBA promoter was produced and purified according to the methods described by Carty et al. [35]. The human α-syn clone was confirmed to be reference number GI:225690599. A dot blot assay was used to determine the viral titer and was expressed as vector genomes (vg)/ml [35]. Animals were injected with 1.5 μL of human α-Syn AAV9 (~1x10^13^ vg/mL) or control GFP virus (~1x10^13^ vg/mL) into the right SN at a flow rate of 2.5 uL/min. The virus was injected using the CED method described previously [35]. Surgery was performed as described previously [36]. Injection coordinates for delivery of the recombinant AAV were the following: anteroposterior: −5.6 mm, lateral: −2.4 mm from bregma, and dorsoventral: −7.2 mm from dura.

### Generation of anti-α-Syn antibodies and intra-peritoneal delivery

Two major B cell epitopes within the full length human α-Syn protein were identified based upon antigenicity analysis using DNASTAR Lasergene software (**Fig. 1**). Peptides spanning these two major B cell epitopes were synthesized by Biomer Technology (CA, USA). The peptides were designated fragments A and B which span the N terminal and central regions of the protein respectivley (sequence shown in Fig. 1C). Each of the peptides (250 μg each) were used to vaccinate six month old female boyer goats in order to generate peptide specific antibodies. A series of 3 injections at 3 week intervals were performed. The first injection was with 500 μg peptide mixed with MPL adjuvant. The two subsequent injections were 250 μg peptide mixed with MPL adjuvant. The inoculated goats were bled two weeks after each inoculation to check the antibody response by standard ELISA methods as described below.

Anti-peptide antibodies were purified by mixing the goat antisera with peptide conjugated magnetic beads (Pierce Biotechnology, Rockford, IL). Beads were generated as per the protocol provided by Pierce Bioechnology. Briefly, the peptide conjugated magnetic beads were incubated with the goat antisera. Beads were concentrated using a magnet and the beads were washed followed by elution of purified antibody from the beads by standard methods [37]. Control non-immune goat IgG was purified for use in the experiments using protein G (PanoAb Inc., Temple Terrace, FL). In the study reported here only antibodies against the AB1 (N terminal) and AB2 (central region) of the protein were used as our primary interest was to examine regions outside of the C-terminus. In future studies we will compare results with the C terminal that has been reported by others [32,38].

Antibodies, either control IgG, AB1 or AB2 were injected intraperitoneally (i.p.) at 1mg/rat in 200 μl PBS for the first two injections, then reduced to 0.5 mg/ml for the subsequent 2 injections, and reduced to 0.25 mg/ml for the final injections. Antibody injections were performed one week after intracranial injection of the AAV9-α-Syn with subsequent administrations every 2 weeks for 3 months. Blood from the treated and control rats was periodically sampled (just prior to injection and one week after each injection) in order to measure, by ELISA, sera antibody levels. These time points for administrations samplings, and analyses are indicated in **Fig. 1**. Antibody levels increased after injections, however, the clearance of antibody increased over time.

### Determination of potential antibody treatment effects on behavioral deficits in α-Syn expressing rats as measured by the paw use bias cylinder test

At one, two and three months following stereotaxic surgical delivery of the AAV9-α-Syn vector or controls, measurement of potential effects of antibody administrations were made using the cylinder test which assesses paw use bias. This test is a straight-forward and valid analysis of unilateral defects in voluntary forelimb use which was originally utilized for the detection of limb impairment in rats with unilateral 6-hydroxydopmaine lesions, another model for PD [39]. Subsequent use of the cylinder test in other rat and mouse models of PD has further confirmed this to be a simple and efficient method for measuring lesions, which affect forelimb use [40–42]. Moreover, this test is designed to score animal paw movements initiated by the animal without influence from the experimenter. As well, this test measures the asymmetry between the affected and unaffected limbs with each animal functioning as its own control for individual differences in forelimb impairments. Briefly, for this test rats were placed in a 4-liter transparent upright Plexiglas cylinder measuring 30 cm high by 20 cm in diameter. The number of either left or right or both forelimb placements on the wall of the cylinder were recorded, and the percentage of each limb or both limbs placements compared to the total limb placements was calculated. At least twenty paw touches per rat were recorded for the analysis.

### Immunohistochemistry and Stereological Quantification

Following behavioral testing rats were divided into two groups with even distribution of paw bias scores. One group was used for biochemical analyses (described below), and the other group was used for immunohistochemistry (IHC). For IHC rats were anesthetized with isoflourane and perfused transcardially with phosphate buffered saline (PBS), followed by treatment with 4% paraformaldehyde in PBS. The brains were carefully removed and postfixed in paraformaldehyde overnight followed by equilibration in 30% sucrose in PBS for at least 24 hours. The brains were then sectioned into 40 μm coronal slices with every sixth section within the SN selected for immunostaining. Tyrosine hydroxylase (TH) activity was measured since this enzyme is the rate limiting step in the generation of dopamine, and is specific for the dopaminergic neurons in the SNc. The diminution of dopamine has been demonstrated to be correlated to α-Syn levels [43]. For immunohistochemical analysis, brain slices were first incubated in sodium periodate (PBS/NaIO_4_) for 20 minutes, then blocked in PBS/0.1% Triton X-100/3% normal goat serum for 1 hr and incubated overnight with the appropriate primary antibodies (mouse anti TH, Immunostar, 1:10,000; OX-6-mouse anti-RT1B 1:750, BD, mouse Anti-NeuN, Millipore (1:100), purified goat polyclonal anti-α-Syn (1:30000 from 1 mg/ml stock). Slices were then washed and incubated for 1 hour in biotinylated secondary antibodies goat-anti mouse or rabbit-anti-goat followed by three washes before one hour incubation in an avidin-biotin substrate (ABC kit, Vector Laboratories). Slices were then incubated in DAB (diaminobenzidine) solution with metal enhancer for OX-6, NeuN, and Syn staining or without metal enhancer for TH staining. Slides were dried, dehydrated through a graded alcohol series into xylene and cover-slipped with permount mounting medium. The Optical Fractionator method of unbiased stereological cell counting was used to estimate the number of TH+, NeuN+, and OX-6+ cells in the SN. The sections were viewed on an Olympus BX-60 microscope (Melville, NY) using a CCD video camera (HV-C20, Hitachi, San Jose, CA). Contours were determined at 2X magnification and cell counting was performed at 40X using the optical fractionator. The sampling site was customized to count at least 200 cells per brain. For counting TH, NeuN, and OX-6 positive cells, the counting frame were 70×70 μm 75×75 μm and 400×300 μm with a virtual counting grid of 140×140 μm, 160×160 μm, and 400×300 μm, respectively. For quantification of α-Syn immunoreactivity, every 6^th^ brain section throughout the region of interest were imaged using a Mirax Scan digital slide scanner (Carl Zeiss USA). The percent area of positive α-Syn staining in the SN slides was quantified using Image analysis software (NearCYTE, http://www.nearcyte.org/) as described previously [35].

### Measurement of brain human α-Syn levels and levels of administered anti-peptide antibodies

For *biochemical analysis*, a separate group of rats (AAV-GFP control [n = 5], AAV α-Syn + IgG [n = 6], AB1 [n = 5], and AB2 [n = 5] were perfused with PBS and brains were *snap-frozen* in liquid nitrogen and stored at −80°C until assayed. α-Syn level detection: A sandwich ELISA kit (Biomer Tech, Inc) was used to determine α-Syn levels in brain tissues. Briefly, a series of 50 μL of α-Syn protein as standards (1250,625,313,156,78,39,19.5 and 0 pg/ml) were added to a 96 well plate pre-coated with goat anti-human α-Syn antibody (250 ng/well). This constituted the standard curve for the analysis. The other wells of the plate had added to them brain lysates (250 μg of protein) samples from the different experimental and control groups. Then 50 μl of rabbit-anti-human alpha synuclein antibody (i.e. detection antibody which had antigen specificity against α-Syn distinct from that of the capture antibody was added into each well and mixed on an orbital shaker. The plate was then incubated for 3 hours at RT. Following the incubation the plates were washed and subsequently incubated at RT with Biomer Tech anti-rabbit AP (alkaline phosphtase) conjugated antibody (1:5000 dilution, 100μl/well). This was followed by another washing and incubation with diluted BioFXUltra Sensitive AP 450 nm solution for 20 min 100 μl/well at a 1:10 dilution. The plate was then subsequently read at 450 nm (for chemiluminescense) with concentration levels calculated based on the standard curve.

**Detection of injected antibody**. Plates were coated with the α-Syn peptides (500 ng of individual peptides per well) used to generate the antibodies and incubated overnight at 4°C. After blocking for 1 hr in 1.5% BSA-PBST, 100 μL/well of the rat plasma samples at a 1:200 diltution in 1.5% BSA-PBST were added and incubated overnight. Normal goat IgG used as a standard in this assay. Following another wash, 100 μL/well of anti-goat IgG-HRP (horseradish peroxidase) (A-9452) in 1.5% BSA-PBST was added and incubated at 37°C for 45 min. The plate was then incubated with BioFX substrate (100 μL/well) for 5 min at RT after several washes and then measured at 450 nm for chemiluminescence.

### Statistical Analysis

One-way analysis of variance (ANOVA) was used for multiple group analysis, with the significance level α = 0.05, was used for statistical analysis, as indicated, for every set of experimental data with the exception of the cylinder test where a two-way repeated measures ANOVA was used. A Bonferroni’s post hoc test was conducted to assess further differences among groups. All values were expressed as mean ± SEM. Graphs were generated, and statistical analyses performed using GraphPad Prism 5.0 (GraphPad Software, La Jolla, CA, USA).

## Results

### Study design to test the efficacy of anti-α-Syn antibodies as passive immunotherapy in an AAV-α-Syn rat PD model

Several animal models have been developed which mimic many of the clinical features of PD. As indicated, it has been demonstrated that injection of an AAV vector expressing WT α-Syn into the SN of rats resulted in a 20% loss of DA neurons 4 weeks after transduction, with a futher progressive loss of the neurons up to 50–60% at 2–6 months post transduction depending on the AAV serotype and amount of vector delivered. Our previous studies with the AAV9-α-synuclein used here demonstrates widespread expression of α-synuclein throughout the substantia nigra associated with 50% cell loss at 2 months post delivery [44–46]. **Fig. 1** summarizes the study design of the experiments presented in this report including: (a) the time frame for antibody delivery, blood collections and analyses and (b) the timing of the behavioral testing (c) sequence of the peptides used to generate the α-Syn specific antisera as well as (d).the kinetics of injected antibody levels. The results shown in (d) demonstrate that antibody levels remained high for at least a month.

### Anti-α-Syn antibodies ameliorate paw use bias post AAV-α-Syn injection

**Fig 2:**
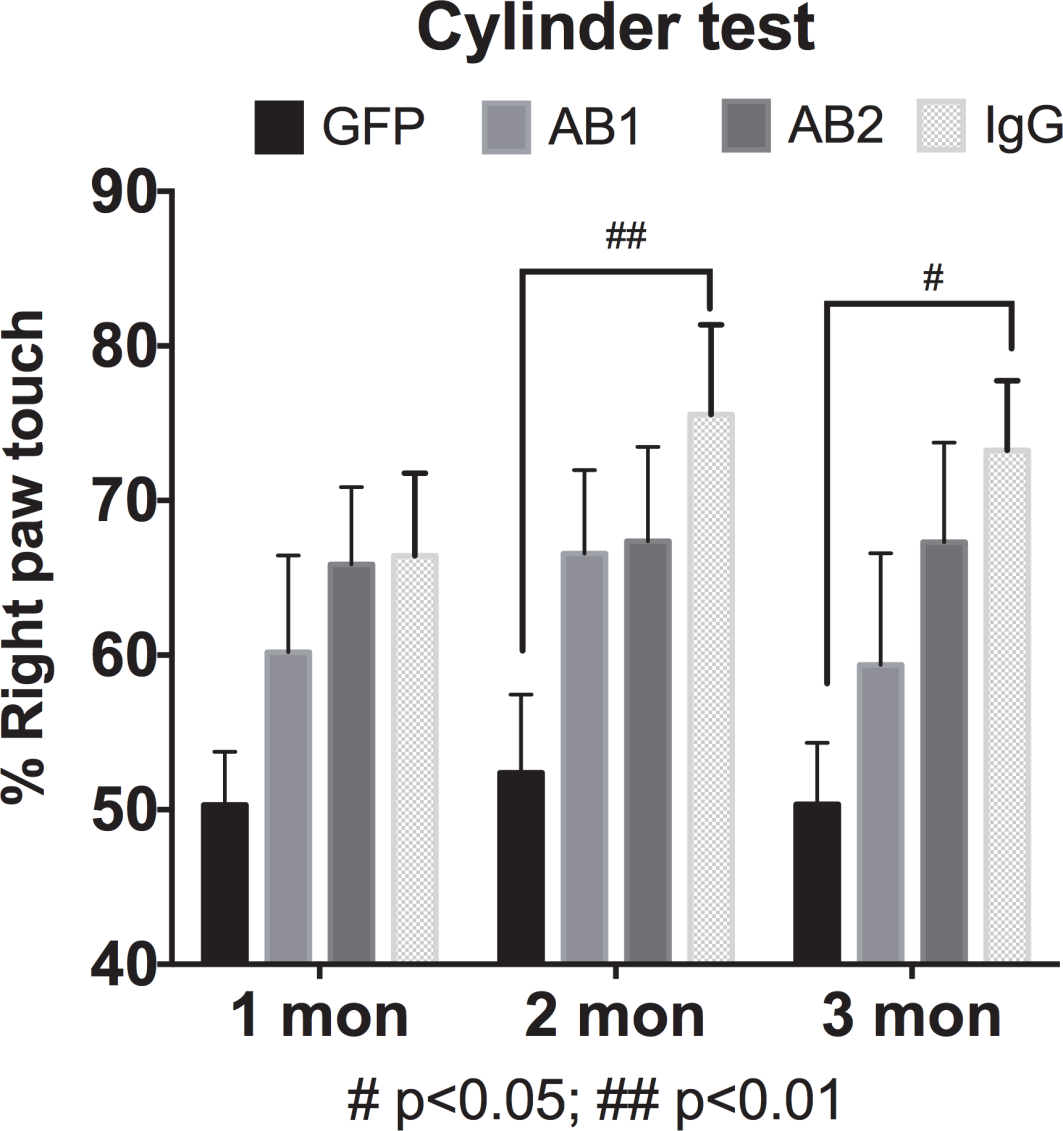
Effect of intraperitoneal administered anti-α-Syn antibodies on motor function. AAV-α-Syn+IgG rats demonstrated paw bias in the cylinder test when compared with AAV-GFP controls starting at two months and continued at 3 months, likely reflecting the progressive nature of this model with ongoing DA cell loss (A two-way ANOVA found a main effect of treatment, F_3,67_ = 4.48, p = <0.001). Although AB1 or AB2 antibody treatment did not demonstrate any significant improvement in behavioral deficits compared to AAV-α-Syn+IgG treated animals, at no time was the AB1 nor AB2 group significantly different from the control AAV-GFP group. Data are presented, as the percent right paw preference ± SEM (n = 23 AAV-GFP control and n = 16 for treatment groups).

The effects of anti-α-Syn antibodies on AAV-α-Syn induced motor deficits, using a cylinder paw preference test was assessed. Significant motor deficits were observed to develop over time in the IgG treated AAV-α-Syn group, compared to the control AAV9-GFP group, consistent with a progressive loss of DA neurons as we and others have observed previously [45,46]. Paw use bias was observed in synuclein treated rats with IgG treatment beginning two months after AAV-α-Syn injection (a two-way ANOVA indicated a treatment effect of α-Syn (F_3,67_ = 4.78, p = <0.001, Bonferonni’s tests p<0.01). The AAV-α-Syn + IgG treated rats continued to remain different from control at both 2 and 3 months (Bonferonni’s test ** *P* <0.01 vs GFP). (**Fig. 2, Table 1**). On the other hand, the AB1 and AB2 treated groups did not demonstrate behavioral impairment that was statistically different from GFP control group during the entire study period (Bonferonni’s test P *>*0.05). The performance score of AB1 treated rats was always closer to 50% than AAV-α-Syn+IgG rats but there was no statistically significant difference between the groups.

**Table 1:** Paw preference for all groups.

Group name	Number of subject	% Right paw preference		
		1 mon	2 mon	3 mon
AAV-GFP/PBS	23	50 ± 3.9	52 ± 4.52	50 ± 3.35
AAV-α-Syn + IgG	16	66 ± 5.50	76 ± 6.00	73 ± 4.64
AAV-α-Syn + AB1	16	60 ± 6.46	67 ± 5.59	59 ± 7.49
AAV-α-Syn + AB2	16	66 ± 5.16	67 ± 6.28	67 ± 6.65

### Treatment with anti-α-Syn antibodies reduces α-Syn levels in the SN

**Fig 3:**
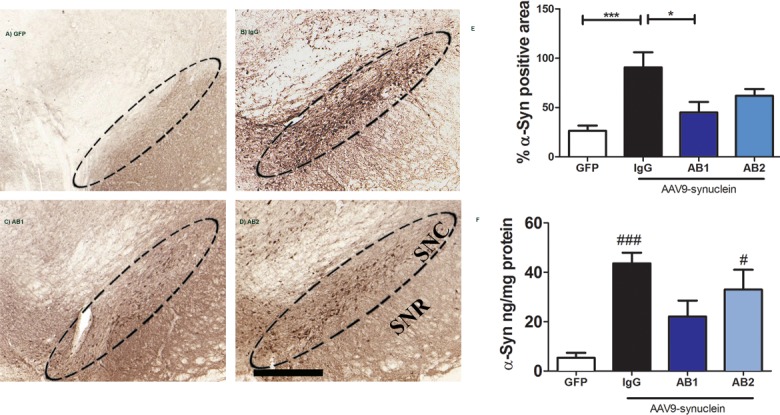
The effect of intraperitoneal administered anti-α-Syn antibodies on AAV vector mediated α-Syn expression. Immunostaining of the SN region with an antibody against α-Syn. Administration of AAV-α-Syn into the rat SN caused significant expression of α-Syn in the SN (B) compared to the AAV-GFP control group (A). Intraperitoneal injection of anti-α-Syn antibody AB1 reduced α-Syn level in the SN (C), while injection with antibody AB2 had a reduced effect (D). Quantitative analysis of levels of α-Syn expression is presented as percent positive area (E). Data are presented as the percent positive area of anti-α-Syn staining throughout the SN (n = 8 animals per group). Asterisk denotes significance (*** P<0.001, * P<0.05) with comparison made to the ipsilateral AAV-GFP group by 1-way ANOVA with post-hoc Bonferroni test. ELISA analysis confirmed a significant reduction in α-Syn levels in the SN with antibody AB1 compared to IgG treatment (F). ### P< 0.001, # P< 0.05 vs. Control AAV-α-Syn + IgG. Data are presented as the mean concentration of α-Syn in pg/μg of protein ± SEM (n = 6 per group). Scale bars are 100μm.

There was extensive expression of α-Syn in and around the AAV injection site at 3 months post AAV injection (**Fig. 3B,E**). Rats treated with anti-α-Syn antibodies demonstrated visibly less positive α-Syn staining in the SN than AAV-α-Syn expressing rats treated with control IgG (**Fig. 3C,D**). Compared to AAV-GFP, injection of AAV-α-Syn significantly increased α-Syn expression in the SN one-way ANOVA (F (3, 27) = 7.215, p = 0.001) (**Fig 3E**). Anti-α-Syn antibody AB1 treatment significantly attenuated α-Syn accumulation compared to AAV-α-Syn + IgG (*p<001). ELISA based measurements using tissue homogenates from ipsilateral SN was used to further confirm the effects of the anti-α-Syn antibodies on α-Syn accumulation. Compared to AAV-α-Syn + IgG, administration of anti-α-Syn antibodies resulted in a significant reduction in α-Syn levels in the SN as analyzed by one-way ANOVA (F (3, 21) = 8.207, p = 0.0012) (**Fig. 3F**). Anti-α-Syn antibody AB1 treatment attenuated extensive α-Syn accumulation by 50% compared to AAV-α-Syn + IgG (p<001).

### Anti-α-Syn antibodies protect against α-Syn induced DA neuron and NeuN positive cells loss in the SN

**Fig 4:**
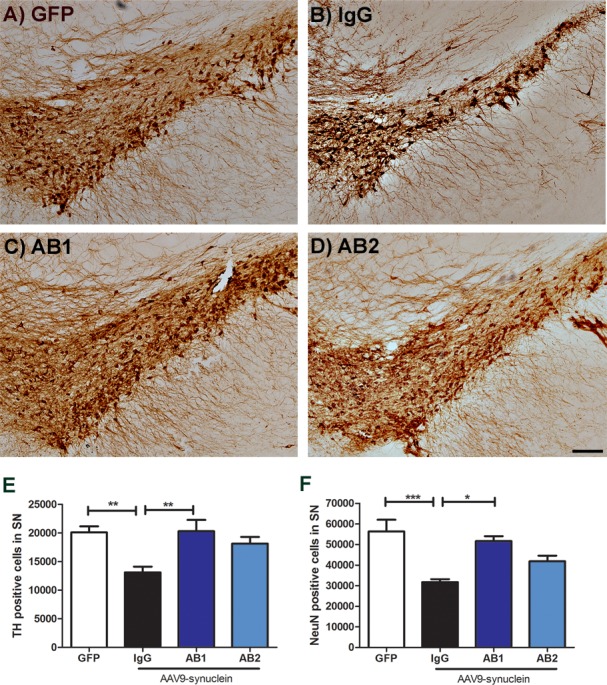
Rescue of TH+ and NeuN+ cells in the ipsilateral SN with intraperitoneal administration of anti-α-Syn antibodies. Immunohistochemical staining of the SN region with an anti-TH antibody (A) AAV-GFP, (B) AAV-α-Syn + IgG, (C) AAV-α-Syn + AB1, (D) AAV-α-Syn + AB2. (E) Graph of unbiased stereological estimation of TH+ cells in the SN of treated animals. AB1 treated animals showed similar levels of TH+ cells compared to the GFP control and significantly higher number of TH+ cells compared to the IgG treated group. Data are shown as mean ± SEM (n = 13 AAV-GFP control and n = 7 for treatment groups). (F) Graph of NeuN+ cells of the SN. Stereologic analysis shows a significant rescue of NeuN+ cells in SN sections with AB1 compared with IgG treated animals (n = 9 AAV-GFP control and n = 7 for treatment groups). *P< 0.05, **P< 0.01, ***P< 0.001. Scale bar = 100μm.

The expression of AAV delivered α-Syn resulted in a 40% loss of TH positive cells in the SN at 3 months in the control AAV-α-Syn + IgG group (**Fig. 4B,E**). Unbiased stereological counting of TH positive neurons demonstrated that anti-α-Syn antibodies AB1 significantly protected neurons from AAV-α-Syn induced toxicity (one-way ANOVA (F (3,30) = 5.8, *p* = 0.002) (**Fig. 4E**). These results demonstrate that the anti-α-Syn antibody AB1 was able to rescue dopaminergic neurons from death due to α-Syn mediated neurodegeneration. Since loss of TH staining can occur without the loss of neurons the number of NeuN positive cells within the SN was examined in each treatment group. Loss of NeuN positive cell counts indicates DA neuron cell death and not the down regulation of TH phenotypic changes. As expected, a significant loss of NeuN+ve neurons was observed in the SN in the AAV-α-Syn + IgG group (one-way ANOVA (one-way ANOVA (F (3, 29) = 7.92, *p* = 0.002) (**Fig. 4F**). As with the TH staining, the decline in the number of NeuN positive cells was not observed with AB1 treatment (**Fig. 4F**). As noted, with the anti-TH staining, AB2 demonstrated an intermediate rescue of NeuN positive cells compared to AB1 (treatment was not statistically different from the control group).

### Anti-α-Syn antibodies reduce microglial activation in the SN

**Fig 5:**
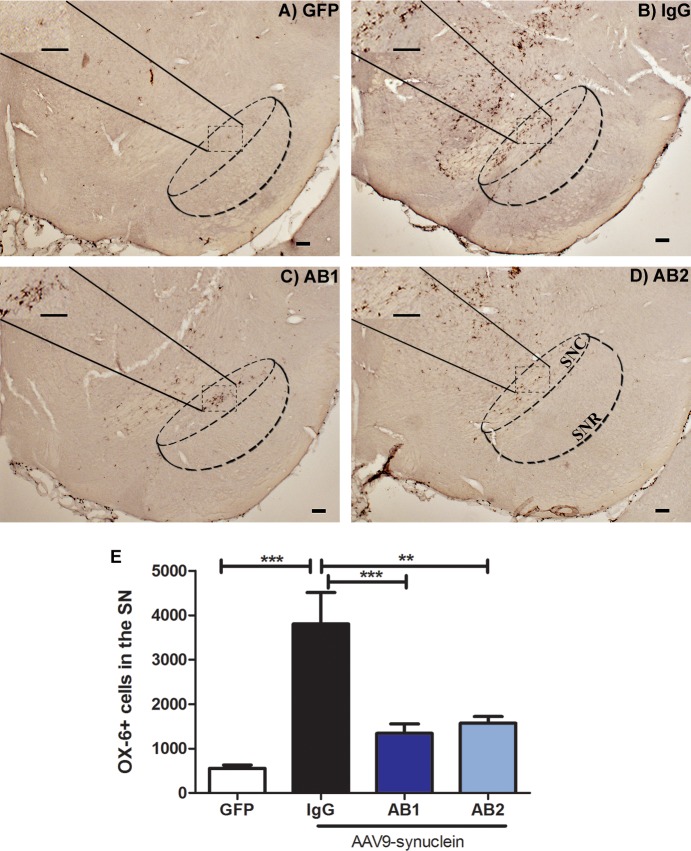
Effect of intraperitoneal administered anti-α-Syn antibodies on the number of OX-6+ cells (MHCII). Micrographs of anti-OX6 staining for (A) AAV-GFP, (B) AAV-α-Syn + IgG, (C) AAV-α-Syn + AB1, and (D) AAV-α-Syn + AB2. Strong immunoreactivity for OX-6 is shown in the inset at high power magnification (40X). Increased OX-6 immunoreactivity was present in AAV-α-Syn + IgG treated rats compared to the other three groups. (E) Graph of unbiased stereological estimation of OX6+ cells in the SN. There is a significant decreased in the number of OX-6+ cells in groups that received anti-α-Syn antibodies (AB1,AB2) compared with the control IgG groups. Data are shown as mean ± SEM. (AAV-GFP control [n = 14], AAV α-Syn + IgG [n = 9], AB1 [n = 8], and AB2 [n = 6] were analyzed) ***P< 0.0001 ** P< 0.001 by one-way ANOVA with post-hoc Bonferroni test. Scale bar = 100μm.

Analysis of the potential effect of α-Syn on activated microglia was made using the monoclonal antibody OX-6 which is directed against MHC II antigen, and as such can be considered to be a marker for microglial activation. Immunostaining revealed OX-6-immunopositive microglia distributed across the ipsilateral SN regions (**Fig. 5**). Activated microglia demonstrated the characteristic bushy morphology with increased cell body size and contracted and ramified processes. There was a significant difference between groups in the numbers of MHCII expressing cells at three months after administration of the anti-α-Syn antibodies. One-way ANOVA revealed an overall effect of anti-α-Syn antibody treatment of the SN (F_(3, 36)_ = 16, *p* = <0.0001). There were more activated microglia in the ipsilateral SN in all of the α-Syn treated groups; however, the numbers were lower in groups treated with the anti-α-Syn antibodies compared to AAV-α-Syn + IgG group (**Fig. 5 B-E**).

## Discussion

In this study, in order to identify a novel epitope to use for vaccine development, we generated several antibodies to major α-Syn epitopes and evaluated targeted passive immunotherapy. Of particular interest were antibodies which were directed against regions not previously tested in an animal model of PD pathology. These were designated AB1 (against N-terminal) and AB2 (against the mid region of α-Syn). We examined an established PD model of AAV mediated over expression of human α-Syn within the rat SN. This model, importantly, induces a progressive PD-like pathology [44,46]. In our control experiment (AAV-α-Syn + non-immune IgG adminstration), a 40% reduction in TH+ cells in the SN was observed, which is consistent with previous reports of this model [44,46] In the experimental groups novel polyclonal antibodies generated against two identified putative B cell epitopes within the N-terminal and central regions of human α-Syn were administered to the α-Syn expressing rats. It was observed that the AB1 anti-α-Syn antibodies could prevent α-Syn induced DA cell loss and improve behavioral outcomes, whereas AB2 was less effective. Rats expressing α-Syn without any anti-α-Syn AB treatment demonstrate significant paw use bias using a cylinder test starting at two months when compared to animals expressing AAV-GFP (controls). Rats expressing α-Syn and treated with the anti-α-Syn AB1 or AB2 antibody showed a trend towards amelioration of this behavioral deficit as they were not significantly different from the control AAV-GFP treatment group at any time point. Taken together, this would suggest that AB1 may be a better target region (N-terminus) as this antibody seems to alter the progression of the overall deficit more effectively than AB2 (central domain).

More importantly, we observed a significant rescue of α-Syn mediated TH+ and NeuN+ neuron loss in the SN after treatment with the AB1 antibody. The AB1 treatment was identical to the GFP control group and statistically different from the IgG treated group. Treatment with the AB2 antibody trended toward an amelioration of neuron loss but was not statistically significant from either the untreated or GFP expressing control groups, again suggesting that the AB1 epitope might be a better target for development of immunotherapies than the AB2 epitope. The intermediate rescue of neuron loss in the AB2 treated animals would suggest that this antibody is less efficacious.

Microglial activation is well established in many neurological diseases including PD. We examined the level of microglial activation with the MHCII marker, OX-6. As observed previously by others and us, we observed a significant increase in OX-6 staining in our α-Syn over expressing model (Fig. 5) [46]. More interestingly, we observed a reduction in the number of activated microglia in α-Syn expressing rats treated with either the AB1 or AB2 antibodies. This reduction is likely due to the decrease in toxic α-Syn species present in the brain and thus the reduction in neuron cell death due to α-Syn.

Recently therapeutic efficacy with three different anti-α-Syn antibodies, directed against the C-terminal region of α-Syn, has been reported [38]. These investigators determined that antibodies induced by the peptide α-Syn_126–140_ immunoprecipitated higher levels of α-Syn from brain extracts than the other two antibodies tested. In contrast to the Ghochikyan et. al. study our group, as indicated, generated antibodies directed against the N-terminal and central regions of human α-Syn. It has been well documented that the N-terminal amino acids are essential for formation of amphipathic alpha-helix responsible for α-Syn membrane recognition [47]. Deletion of N-terminal amino acids have shown not only decreases in the helix propensity of α-Syn but also reductions in the toxicity of α-Syn protein in yeast [48]. This finding implies that the N-terminal amino acids may initiate the folding of the entire α-Syn protein and promote formation of toxic α-Syn protein. As indicated above, both of the anti-α-Syn antibodies generated in our study reduced the α-Syn level within the brain. However, our N-terminal targeted AB1 antibody proved more efficacious than the central domain epitope (AB2). During the preparation of this manuscript a report was published which demonstrated that an antibody against N-terminus amino acids 1–5 of α-Syn has the ability to reduce pathology in α-Syn mouse model [33]. This epitope is distinct from the region studied in this report. Thus, targeting the N-terminal region of α-Syn protein might represent a useful immunotherapeutic approach to treat PD as well as other α-synucleinopathies.

There are several potential mechanisms for antibody mediated clearance of α-Syn. Firstly, the peripheral sink hypothesis proposes a shift of the α-Syn equilibrium from the central nervous system to the peripheral blood. This is based on the now well established observation that there is a dysfunctional blood brain barrier (BBB) in PD patients [49]. In this model abnormal α-Syn protein is envisioned to enter into the peripheral circulation through a “leaky” BBB with subsequent activation through antigen-presenting cells resulting in the induction of adaptive immune responses. Thus, these antibodies may exert their effect in the brain by reducing the levels of α-Syn load in the periphery which, in turn, reduces the accumulation of α-Syn in the brain [50]. Second, an alternative mechanism for clearance of α-Syn by anti-α-Syn antibodies may involve the formation of extracellular immune complexes with secreted α-Syn leading to microglial activation. It has been determined that the MHCII protein is critical for α-syn induced microglial activation, IgG deposition, and CD4 T cell proliferative responses [51]. In fact, it has been demonstrated that that MHCII knockout mice are protected from α-Syn induced dopaminergic neurodegeneration [51]. Eun-Jin Bae and colleagues recently employed a passive immunotherapy approach to test the efficacy of anti-α-Syn antibodies in the clearance of extracellular α-Syn aggregates in microglial cells [32]. These authors demonstrated that these anti-α-Syn antibodies block cell to cell transfer of extracellular α-Syn by promoting α-Syn acquisition into microglia, which were then delivered to the lysosome for destruction. Targeting extracellular α-Syn may reduce the likelihood of adverse functions of proteins inside the neuronal cytoplasm and would be a unique approach for treating the consequences of abnormal α-Syn deposition. But microglial activation and functional shifting are questions that remain to be resolved. Guo and Lee has reported that α-Syn possesses cell-to-cell transmission ability. They hypothesize that functional antibodies may reduce pathology, at least in part, by inhibiting this cell transmission process [33,52]. Our group has also demonstrated that the function of microglia is restored after antibody treatment, potentially allowing microglia to more effectively digest α-Syn to prevent disease progression.

Vaccine and immunotherapy strategies have been determined to be effective in animal models of neurodegenerative diseases such as PD and AD, but have failed in clinical trials targeting specifically Aβ in AD, due to both safety and efficacy concerns [53,54]. Although not completely without drawbacks the passive immunotherapy may have several advantages. Firstly, the dose can be controlled by monitoring blood antibody levels with subsequent cessation of treatment if there are any adverse reactions. Secondly, a cocktail of different epitope specific antibodies may offer a more effective therapy. Despite these advantages, certain issues need to be overcome in order to assess their long-term clinical safety and efficacy. To ensure adequate amounts of efficacious antibodies in the brain, these antibodies need to cross the blood brain barrier easily [55]. A second major hurdle would be that repeated injection of antibodies over time may lead to the formation of antibodies against the previously administered antibody which could potentially neutralize their potentially beneficial effects and/or lead to serious side effects [56]. Assessment of the risks associated with the use of these polyclonal antibodies need to be evaluated, and necessary steps taken to identify and minimize potential adverse effects. One potential method for reducing adverse effects would be the use of humanized antigen specific monoclonal antibodies for use as therapeutic agents. In order to obtain an optimal therapeutic effect, these antibodies may require adiminstration prophylactically at earlier onset of PD pathological changes. Further, studies comparing therapeutic effects of anti-N-terminal antibodies with other antibodies that recognize other regions of α-syn or post-translational modifications of the protein are needed to confirm the potential of these novel antibodies for PD treatment.

## Conclusions

In recent years, the passive immunotherapy approach has been an attractive potential strategy for the treatment of PD. Our data further support that passive immunotherapy targeting α-Syn might be a possible therapeutic approach for slowing the progression of symptoms of PD. Further, our data suggest that targeting of the N-terminal domain would be a more effective treatement than targeting the central domain of α-Syn. This may be because the N-terminal domain is more structured and therefore offers a better therapeutic target than other regions of the α-Syn protein. However, additional research is warranted to evaluate the mechanism by which active or passive vaccination against α-Syn mediates clearance of this protein from the brain as well as ameliorating the pathological mainfections of PD including those affecting behavior.
